# Combined Use of Serum N-terminal Pro-B-Type Natriuretic Peptide and Glypican-6 in the Diagnosis of Heart Failure

**DOI:** 10.7759/cureus.45766

**Published:** 2023-09-22

**Authors:** Emre Cem Sağlam, Metin Yadigaroğlu, Murat Güzel, Hatice Turan, Şakir Hakan Aksu, Metin Ocak, Selim Gorgun, Uğur Arslan, Murat Yücel

**Affiliations:** 1 Emergency Medicine, Sadıka Sabancı State Hospital, Sakarya, TUR; 2 Emergency Medicine, Samsun University Faculty of Medicine, Samsun, TUR; 3 Emergency Medicine, Kulu State Hospital, Konya, TUR; 4 Emergency Medicine, Samsun Education and Research Hospital, Samsun, TUR; 5 Microbiology, University of Health Sciences, Samsun Education and Research Hospital, Samsun, TUR; 6 Cardiology, Samsun University Faculty of Medicine, Samsun, TUR

**Keywords:** combined use, bnp, nt-probnp, gpc-6, glypicans, heart failure

## Abstract

Objective: The aim of this study was to investigate the efficacy of serum glypican-6 (GPC-6) levels and the combination of N-terminal pro-B-type natriuretic peptide (NT-ProBNP) and GPC-6 in the diagnosis of heart failure (HF).

Methods: In this prospective study, patients older than 18 years of age, admitted to the emergency department of our hospital between December 2021 and April 2022, diagnosed with heart failure (patient group), and healthy volunteers with similar sociodemographic characteristics (control group) were included. The disease severity classification of the patient group was made according to the 2021 ESC guidelines, using echocardiographic findings. Serum GPC-6 and NT-ProBNP levels were measured by the enzyme-linked immunosorbent assay (ELISA) method, which determines the antigen-antibody relationship. Optimal GPC-6 and NT-ProBNP levels for the diagnosis of HF were determined by receiver operating characteristic (ROC) analysis. The patients were divided into three groups according to these levels. Group 1 consisted of patients with both markers below the cutoff values, Group 2 consisted of patients with either of these markers above the cutoff values, and Group 3 consisted of patients with both markers above the cutoff values.

Results: The study included 65 heart failure patients and 20 healthy volunteers. When the patient and control groups were compared in terms of serum GPC-6 and serum NT-ProBNP levels, both parameters were evaluated as significantly higher in the patient group (p=0.038 and p<0.001; respectively). In the ROC analysis, it was determined that GPC-6 indicated HF with 58.46% sensitivity and 75% specificity for an optimal cutoff value of 390 pg/ml. In the ROC analysis, it was determined that serum NT-ProBNP indicated HF with 89.23% sensitivity and 70% specificity for an optimal cutoff value of 122 pg/ml. When the groups were compared according to the rate of HF, it was found to be higher in Group 3 compared to Group 2 (97.1% vs. 70.3%, p<0.002) and Group 1 (97.1% vs. 38.5%, p<0.001). This rate was seen to be significantly higher in Group 2 compared to Group 1 (70.3% vs. 38.5%, p=0.042).

Conclusion: The combination of GPC-6 and NT-ProBNP may help diagnose HF patients admitted to the emergency department.

## Introduction

Heart failure (HF) is a syndrome characterised by the inability of the heart to pump enough blood and oxygen to meet the metabolic demands of other organs [1]. HF is a syndrome that is common worldwide and affects approximately 60 million patients. The aging of the population and the presence of comorbidities worsen the clinical picture, and the prevalence of HF also increases due to the prolongation of survival after myocardial infarction [2,3]. Early diagnosis and clinical categorization are crucial for effective treatment optimization and prognosis improvement in HF. Acute HF or decompensated HF is a common reason for admission to emergency departments. Diagnosing HF can prove difficult, particularly in patients with dyspnea of unknown origin, advanced age, and other comorbidities [4]. There are no specific physical examination findings, electrocardiographic (ECG) findings or imaging modalities that can identify or exclude HF as a cause of dyspnea. The diagnosis of HF is traditionally made by ECG, thorax radiography, clinical examination findings and echocardiography (ECHO) [5].

In previous studies, researchers have focused on biochemical biomarkers in the diagnosis of HF [1,3-9]. The most well-known and most commonly used of these markers are natriuretic peptides. Brain natriuretic peptide (BNP) is synthesized and secreted mainly in response to left ventricular (LV) myocytes stretched by pressure overload or volume expansion of the ventricle [8]. The biological functions of BNP include various compensatory mechanisms involved in HF, including natriuresis, diuresis and vasodilation [9]. N-terminal pro-B-type natriuretic peptide (NT-proBNP) is an inactive metabolite of BNP and circulating NT-proBNP levels are also increased in pathologies that increase BNP [3]. Previous studies have reported that BNP and NT-proBNP are markers with high sensitivity and specificity in the diagnosis of HF and prognosis [1,3-6]. However, it has also been reported that natriuretic peptides give 14-29% false results in patients presenting with dyspnea, even when used in combination with clinical classifications and tests [10-12].

Different studies have emphasized that cardiac remodeling is effective in the development and prognosis of heart failure [13,14]. The extracellular matrix (ECM) is a key stakeholder in this reorganization process. The ECM appears to coexist with many different levels of components, in which proteoglycans also play an important role. Proteoglycans form the structural component of tissue organization. Thanks to this feature, it plays a role in many biological processes such as cell proliferation, adhesion and migration at the vital level [15]. Membrane proteoglycans such as glypicans and syndecans contain mostly heparan sulfate chains in their structure. Glypicans regulate cellular responses to growth factors, which regulate cell migration, differentiation and proliferation, as well as fibroblast growth factor signaling, particularly in relation to remodeling of the cardiac ECM [15]. There are six known forms of glypicans and there are few studies in the literature investigating the relationship between glypican-6 (GPC-6) and HF.

The aim of this study was to investigate the efficacy of serum GPC-6 levels and the combination of NT-proBNP and GPC-6 in the diagnosis of HF.

## Materials and methods

In this prospective study, it was planned to include adult patients, non-pregnant patients and patients without hematological-oncological disease who were admitted to the emergency department of our hospital between December 2021 and April 2022, diagnosed with heart failure (patient group), and healthy volunteers (healthy relatives of patients admitted to the emergency department) with similar sociodemographic characteristics (control group). The patients were diagnosed with heart failure through ECHO (Philips HD11xe Ultrasound, Eindhoven, Netherlands; 2.8-4 MHz phased array probe) that was carried out by the cardiologist on duty, independent of the study. All individuals who participated in the study and/or their legal guardians were informed about the study and their written informed consent was obtained. The ethical approval for the study was obtained from the local ethics committee (Health Sciences University Samsun Training and Research Hospital Non-Interventional Clinical Research Ethics Committee, no:2021/20/21). Our study was conducted in accordance with the Declaration of Helsinki, Good Clinical Practice and Good Laboratory Practice.

The study excluded patients under immunosuppressive therapy, those who presented with intoxication, those who suffered an ischemic stroke during admission, those who had a septic condition, and those with chronic obstructive pulmonary disease/interstitial lung disease.

When the patients were admitted to the emergency department, their required laboratory tests results, imaging results (chest x-ray, chest computed tomography, echocardiography, etc.), vital signs, demographic data, clinical scoring results related to HF and disease severity level results were recorded on a data sheet.

Optimal cutoff levels at the diagnostic stage for both GPC-6 and NT-ProBNP were determined. Patients were divided into three groups according to these cutoff levels. Group 1 consisted of patients with both markers below the cutoff values, Group 2 consisted of patients with either of these markers above the cutoff values, and Group 3 consisted of patients with both markers above the cutoff values.

The disease severity classification of the participants was performed according to the 2021 ESC guideline, using ECHO findings [3]. According to this, the classification was made as follows: 1. HFrEF (Heart Failure with reduced Ejection Fraction): low left ventricular ejection fraction (LVEF), ≤40%; 2. HFmrEF (Heart Failure with mildly reduced Ejection Fraction): mildly reduced LVEF, 41%-49; and 3. HFpEF (Heart Failure with preserved Ejection Fraction): preserved LVEF, ≥50%.

For measurement of serum GPC-6 levels, enzyme-linked immunosorbent assay (ELISA) kit (Wuhan Fine Biotech, Wuhan, China) was applied for in vitro quantitative determination of GPC-6 concentrations in serum.

For measurement of serum NT-ProBNP levels, ELISA kit (Elabscience, Houston, TX, USA) was applied for in vitro quantitative determination of human NT-proBNP concentrations in serum.

Data were analyzed using SPSS Statistics 25 (IBM Corp., Armonk, NY, USA) and MedCalc (version 20; MedCalc Software, Ostend, Belgium) software packages. Categorical variables were expressed as frequencies and percentages, and numerical data were expressed as mean standard deviation or median (minimum-maximum) according to their conformity to the normal distribution. Student's t-test was used for comparison of numerical data showing normal distribution, the Mann-Whitney U test was used for comparison of data not showing normal distribution, and the Chi-square test and/or post hoc test was used for comparison of categorical data. The Kruskal-Wallis test was used for comparisons of numerical data forming more than two groups. The best GPC-6 and NT-ProBNP cut-off values, the area under the curve (AUC), 95% confidence intervals (CI), sensitivity and specificity values were determined by receiver operating characteristics (ROC) analysis. p<0.05 was considered statistically significant

## Results

The study included 65 heart failure patients (patient group) and 20 healthy volunteers (control group). 53.8% (n=35) of the patients were female and 46.2% (n=30) were male. In the control group, 55% (11) were female and 45% (n=9) were male. The mean age of the patient group was 72±11 years and the mean age of the control group was 68±12 years. Both groups were similar in terms of sex and age (p=0.928 and p=0.173, respectively).

The patient and control groups were compared in terms of serum GPC-6 and serum NT-ProBNP levels. Accordingly, both markers were significantly higher in the patient group (p=0.038 and p<0.001; respectively) (Table 1).

**Table 1:** Comparison of GPC-6 and NT-ProBNP Levels of Patient and Control Groups* GPC-6: glypican-6, NT-ProBNP: N-terminal pro-B-type natriuretic peptide * Results were presented as median (min-max) and p<0.05 was considered statistically significant

	Patient Group Median (Min-max)	Control Group Median (Min-max)	P Values
GPC-6 (pg/ml)	457 (140-2774)	251.50 (145-1084)	0.038
NT-ProBNP (pg/ml)	445 (100-2384)	111 (101-580)	<0.001

Serum GPC-6 and serum NT-ProBNP levels and ECHO findings of the patient group were compared. There was no significant correlation between serum GPC-6 levels and severity of the disease (p=0.425). However, a significant correlation was found between serum NT-ProBNP levels and severity of the disease (p<0.001) (Table 2).

**Table 2:** The Relationship Between GPC-6 and NT-ProBNP Levels and the Severity of the Disease* GPC-6: glypican-6, NT-ProBNP: N-terminal pro-B-type natriuretic peptide, EF: ejection fraction, HFrEF: heart failure with reduced ejection fraction, HFmrEF: heart failure with mildly reduced ejection fraction, HFpEF: Heart failure with preserved ejection fraction * Results are presented as n (%) and median (min-max). p<0.05 was considered statistically significant

	Echocardiography Findings	n (%)	Median (Min-max)	P Values
GPC-6 (pg/ml)	HFrEF (EF ≤ %40)	23 (35.4)	483 (141-2443)	0.425
	HFmrEF (EF = %41-%49)	20 (30.8)	657 (140-2774)	
	HFpEF (EF ≥ %50)	22 (33.8)	392.50 (143-1514)	
NT-ProBNP (pg/ml)	HFrEF^a^ (EF ≤ %40)	23 (35.4)	630 (280-2272)	<0.001
	HFmrEF^b^ (EF = %41-%49)	20 (30.8)	364.50 (130-1470)	
	HFpEF^c^ (EF ≥ %50)	22 (33.8)	300 (100-2384)	

**Figure 1:**
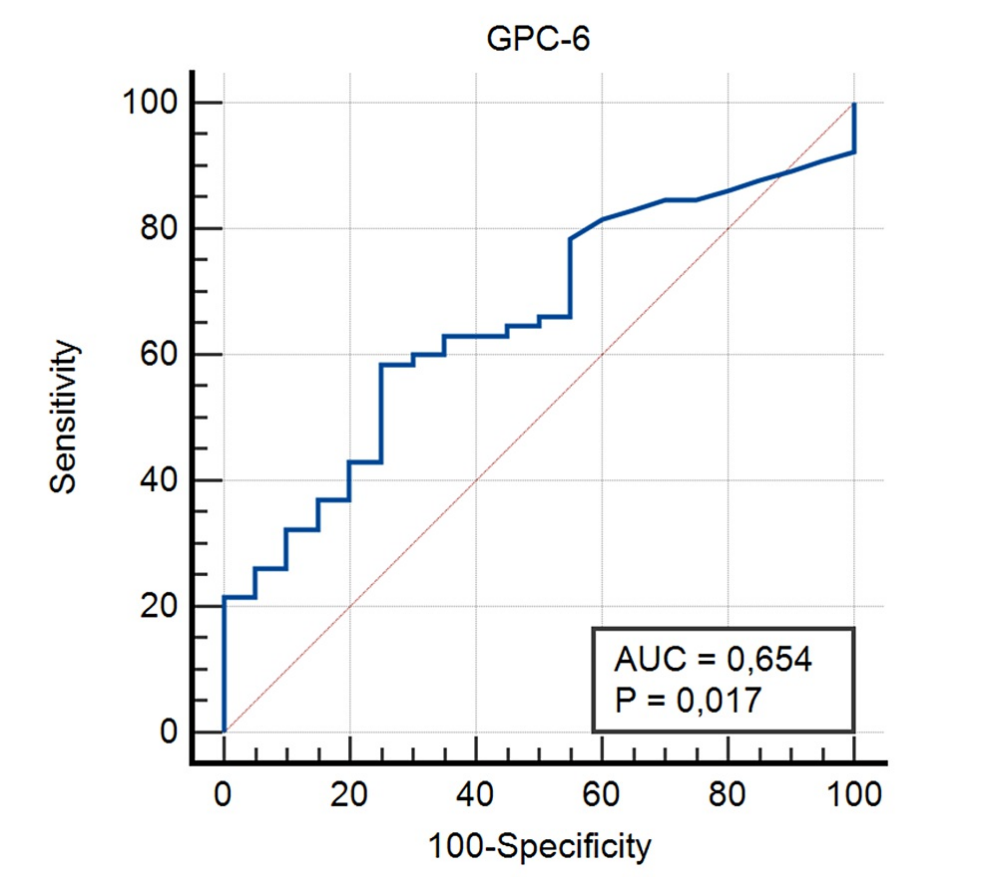
ROC Curve for Serum GPC-6 GPC-6: glypican-6, ROC: receiver operating characteristics, AUC: area under the curve

The sensitivity and specificity of serum GPC-6 and serum NT-ProBNP levels were statistically significant for patient and control groups in ROC analysis (p=0.017 and p<0.001; respectively). In the ROC analysis, the optimal cutoff value of GPC-6 for the diagnosis of HF was 390 pg/ml with 58.46% sensitivity and 75% specificity (Figure 1, Table 3).

**Table 3:** ROC Analysis Results for GPC-6 and NT-ProBNP* GPC-6: glypican-6, NT-ProBNP: N-terminal pro-B-type natriuretic peptide, ROC: receiver operating characteristics * p<0.05 was considered statistically significant

	GPC-6	NT-ProBNP
Area Under the Curve	0.65	0.79
95% Confidence Interval	0.54-0.75	0.69-0.87
Cutoff Value	390 pg/ml	122 pg/ml
Sensitivity	58.46	89.23
Specificity	75	70
P Values	0.017	<0.001

**Figure 2:**
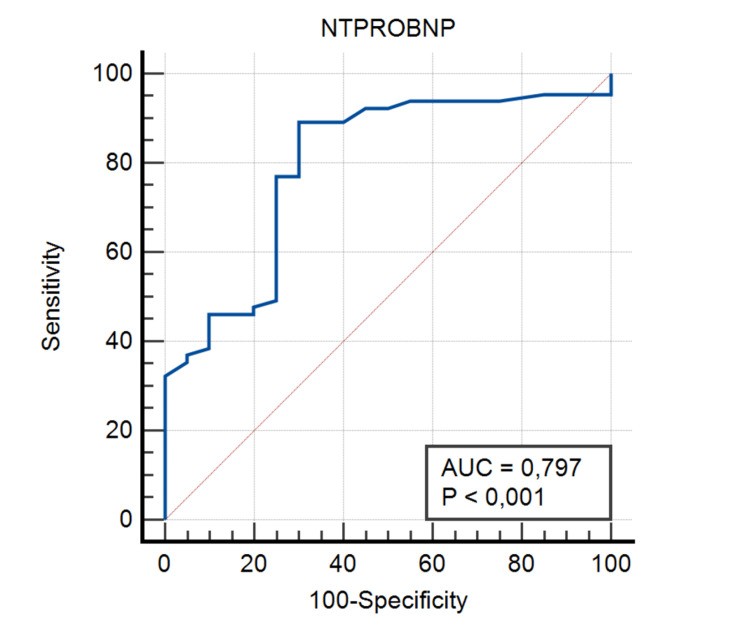
ROC Curve for Serum NT-ProBNP NT-ProBNP: N-terminal pro-B-type natriuretic peptide, ROC: receiver operating characteristics, AUC: area under the curve

In the ROC analysis, the optimal cutoff value of serum NT-ProBNP for the diagnosis of HF was determined as 122 pg/ml with 89.23% sensitivity and 70% specificity (Figure 2, Table 3).

Furthermore, regression analysis revealed that the combination of GPC-6 and NT-ProBNP were independent predictors of HF (Table 4).

**Table 4:** Multivariate Regression Analysis Showing Independent Predictors of HF*** GPC-6: glypican-6, NT-ProBNP: N-terminal pro-B-type natriuretic peptide, HF: heart failure * GPC-6 or NT-ProBNP positive ** GPC-6 and NT-ProBNP positive (combine) ***p<0.05 was considered statistically significant

	Multivariate regression analysis	
Variables	OR (95% CI)	P
Age	1.020 (0.973-1.069)	0.408
Sex	0.665 (0.198-2.238)	0.510
Group 2*	0.098 (0.009-1.049)	0.055
Group 3**	0.070 (0.008-0.573)	0.013

**Figure 3:**
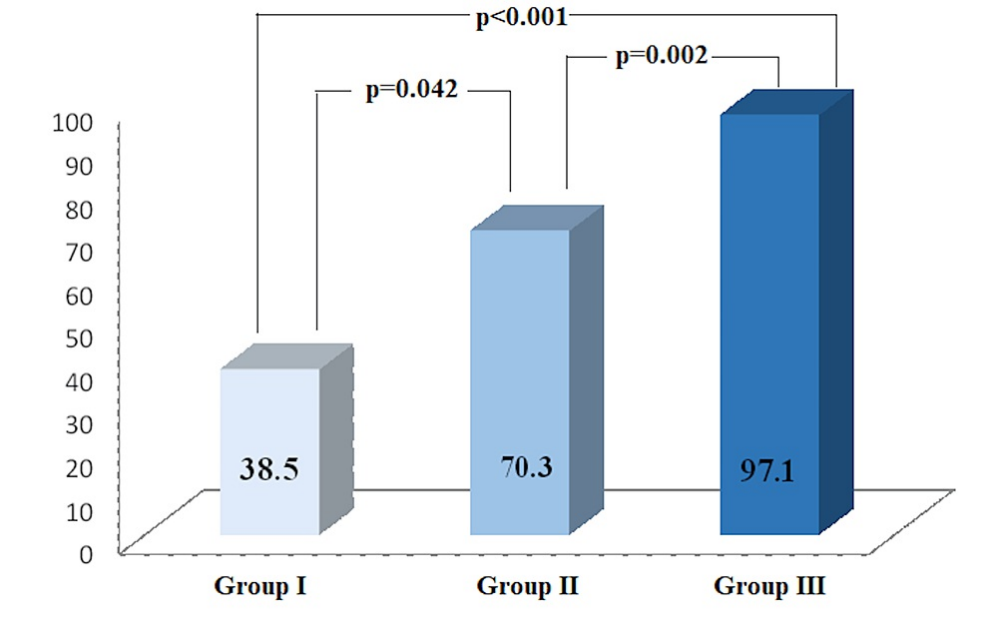
Comparison of Groups Showing Heart Failure Percentages

According to the cutoff values determined by ROC analysis, the patients were divided into three groups. There was a total of 13 participants in Group 1 (NT-ProBNP<122 pg/ml and GPC-6<390 pg/ml), 37 participants in Group 2 (NT-ProBNP<122 pg/ml and GPC-6 >390 pg/ml or NT-ProBNP>122 pg/ml and GPC-6 <390 pg/ml) and 35 participants in Group 3 (NT-ProBNP>122 pg/ml and GPC-6 >390 pg/ml). It was determined that the heart failure rate was higher in Group 3 compared to Group 2 (97.1% vs 70.3%, p<0.002) and Group 1 (97.1% vs 38.5%, p<0.001). This rate was significantly higher in Group 2 than in Group 1 (70.3% vs. 38.5%, p=0.042) (Figure 3).

## Discussion

The findings of our study showed that in patients with HF admitted to the emergency department, the combined use of GPC-6 and NT-ProBNP indicates HF at higher rates than the use of these two parameters separately. We also found that GPC-6 indicated heart failure with 58.46% sensitivity and 75% specificity. In addition, we showed that the combination of GPC-6 and NT-ProBNP were independent predictors of HF. To our knowledge, this is the first study that investigated combined use of GPC-6 and NT-ProBNP in the diagnosis of HF. It is also one of the rare studies that investigated the diagnostic value of GPC-6 in HF. We think that this study and its findings will make important contributions to the literature.

The pumping function of the heart is affected if there is a primary deterioration in myocardial contractility and/or an excessive increase in the hemodynamic load on the ventricle. It is observed that a number of compensatory mechanisms develop at different levels to maintain this pump function. The functioning of the volume-pressure relationship in the ventricle and the activation and restructuring of neurohormonal systems can be counted among these important mechanisms. The first two mechanisms come into play in the early period following severe myocardial damage. In addition, it can provide a normal level of pump function. On the other hand, cardiac remodeling begins earlier and this clinical course continues for weeks to months [13]. This adaptation mechanism is vital in the adaptation process after myocardial injury. These changes can be counted as stimuli such as mechanical tension force in myocytes, neurohormones, release of inflammatory cytokines, growth factors and reactive oxygen products [16,17].

Glypicans are considered a family of heparan sulfate proteoglycans that bind to the outer surface of the cell membrane [18]. GPC-6 is the most recently identified member in glypicans family. It has also been associated with different diseases, including cancer, similar to other glypicans [19]. There are few studies in the literature that investigated the relationship between HF and GPC-6. In a previous experimental study, it was reported that GPC-6 levels were elevated in mice with advanced HF induced by aortic banding and overload. The researchers also reported that GPC-6 messenger RNA levels were increased in LV myocardium obtained from explanted heart tissues of patients with end-stage dilated HF and reduced LVEF [20]. Another recent study reported that serum GPC-6 levels were significantly increased in patients who developed HF after ST-elevated myocardial infarction. In addition, in the study mentioned above, serum GPC-6 was shown to indicate HF with 69% sensitivity and 67% specificity at an optimal cutoff value of 7.47ng/ml [13]. In our study, consistent with the literature, we showed that serum GPC-6 levels were significantly increased in patients with HF. We also found that serum GPC-6 indicates HF with 58.46% sensitivity and 75% specificity for an optimal cutoff value of 390 pg/ml.

BNP and NT-proBNP are synthesized from a 134 amino acid pre-hormone encoded by the NPPB gene. After a deposit of 26 amino acids is split, BNP1-108 is produced, which is converted by furin or corin enzymes to BNP1-32, a biologically active molecule, and NT-proBNP1-76, its inactive N-terminal fragment. BNP is primarily produced by ventricular cardiomyocytes in response to volume or pressure overload [3]. BNP and NT-proBNP levels in the circulation are normally very low. But as a mechanism to restore normal hemodynamics, their level is significantly increased in HF patients. BNP promotes arterial vasodilation, diuresis and natriuresis, exerts anti-hypertrophic and anti-fibrotic effects and counteracts activation of the renin-angiotensin-aldosterone system, sympathetic nervous system and endothelin systems. NT-proBNP has a longer half-life (120 versus 20 minutes) and higher plasma concentrations (approximately six-fold) than BNP due to its different clearance [3,5,21].

In the literature, there are many studies that investigated BNP/NT-proBNP levels in heart failure patients. In these studies, it has been reported that BNP/NT-proBNP is a valid parameter as an indicator of prognosis with high sensitivity and specificity in the diagnosis of HF and follow-up of treatment [1,3-6,8-12,21]. The American Heart Association (AHA) also recommends the use of BNP/NT-proBNP in heart failure management in the 2022 AHA/ACC/HFSA heart failure guideline [22]. In our study, we found that NT-proBNP was significantly higher in patients with HF and correlated with the severity of the disease. We also found that NT-proBNP indicates heart failure with 89.23% sensitivity and 70% specificity for an optimal cutoff value of 122 pg/ml. Our findings support the literature in this context.

Previous studies have shown that the combined use of two parameters may have a stronger therapeutic effect and predictive value than the use of either parameter separately. A previous study reports that using P-wave dispersion (PWD) and troponin I together have greater diagnostic efficacy in predicting recurrence in those with paroxysmal atrial fibrillation than using these parameters separately [23]. Another study reported that the combined use of hyperbaric oxygen and N-acetylcysteine in traumatic spinal cord injury is more effective than using these two treatments separately [24]. According to the results of our study, the combined use of GPC-6 and NT-ProBNP indicates HF at higher rates than the use of these two parameters separately.

The fact that our study was performed in a single center and the relatively small number of patients are the main limitations of our study. Studies involving many patients have established the cut-off value of NT-ProBNP for diagnosing HF in the literature. However, due to the small number of HF patients who participated in this study, a different cut-off value for NT-ProBNP emerged from the literature. This is also considered a limitation of the study. Another limitation of this study is that the ECHO performed to diagnose HF in the patients was performed by different cardiologists. This is why the LVEF measurement method could not be standardized. In the future, further studies without these limitations could provide additional support for our findings.

## Conclusions

GPC-6 and NT-ProBNP levels are significantly increased in HF patients. The specificity of GPC-6 was higher than NT-ProBNP in the diagnosis of cardiac HF at optimal cut-off values. However, the sensitivity of GPC-6 was lower than NT-ProBNP in the diagnosis of cardiac HF at optimal cut-off values. Our findings support the use of GPC-6 as a diagnostic test in the diagnosis of HF. The combined use of GPC-6 and NT-ProBNP in these patients indicates HF at higher rates than the use of these two parameters separately. The combination of GPC-6 and NT-ProBNP may help diagnose HF patients admitted to the emergency department.
